# A Supplement with Bromelain, *Lentinula edodes*, and Quercetin: Antioxidant Capacity and Effects on Morphofunctional and Fecal Parameters (Calprotectin, Cortisol, and Intestinal Fermentation Products) in Kennel Dogs

**DOI:** 10.3390/vetsci10080486

**Published:** 2023-07-27

**Authors:** David Atuahene, Annalisa Costale, Elisa Martello, Alessandro Mannelli, Elisabetta Radice, Davide Giuseppe Ribaldone, Biagina Chiofalo, Bruno Stefanon, Giorgia Meineri

**Affiliations:** 1 Department of Veterinary Sciences, School of Agriculture and Veterinary Medicine, University of Turin, 10124 Grugliasco, Italy; david.atuahene@unito.it (D.A.); alessandro.mannelli@unito.it (A.M.); giorgia.meineri@unito.it (G.M.); 2 Department of Science and Pharmaceutical Technology, University of Turin, 10100 Turin, Italy; annalisa.costale@unito.it; 3 Division of Epidemiology and Public Health, School of Medicine, University of Nottingham, Nottingham NG5 1PB, UK; 4 Department of Surgical Sciences, Medical School, University of Turin, 10100 Turin, Italy; elisabetta.radice@unito.it; 5 Department of Medical Sciences, University of Turin, 10100 Turin, Italy; davidegiuseppe.ribaldone@unito.it; 6 Department of Veterinary Sciences, University of Messina, 98122 Messina, Italy; biagina.chiofalo@unime.it; 7 Department of Agrifood, Environmental and Animal Science, University of Udine, 33100 Udine, Italy; bruno.stefanon@uniud.it

**Keywords:** antioxidant, healthy, canine, fecal parameters, microbiome, nutraceutical

## Abstract

**Simple Summary:**

In this study, we tested the antioxidant capacity of three natural products (quercetin, bromelain, and *Lentinula edodes*) and their combination. A new feed supplement based on the combination was formulated and evaluated in a randomized control trial in kennel adult female dogs. The three ingredients tested together showed a high antioxidant capacity. Additionally, the new formulation showed anti-inflammatory and immunomodulatory effects, indicating a potential for use in improving gastrointestinal health and psycho-physical conditions in dogs.

**Abstract:**

Oxidative stress causes several pathological conditions in humans and animals, including gastrointestinal disorders. The aim of this study was to analyze the antioxidant capacity of three natural powdered raw materials containing quercetin, bromelain, and *Lentinula edodes* and develop a new feed supplement for dogs using a combination of them. The total phenolic content (TPC), antioxidant activity, DPPH (2,2-diphenyl-1-picrylhydrazyl), and ABTS (2,2′-Azino-bis (3-ethylbenzothiazoline-6-sulfonic acid) diammonium salt) of the extracts, either individually or in combination, were evaluated colorimetrically. The effects of this supplement on healthy adult dogs’ nutritional, inflammatory, and stress status were evaluated. American Staffordshire Terrier adult female dogs (*n* = 30) were randomly assigned to a control (*n* = 15) or a treated (*n* = 15) group. The supplement was added as powder to the food of the treated dogs once daily for 28 days. There was no significant difference in the body weight and body condition scores between the initial and final phases of the experiment. At the end of our study, a significant decrease in fecal calprotectin, cortisol, indole/skatole, and N-methylhistamine and a significant increase in short-chain fatty acids were observed as compared to the control group. In conclusion, this natural feed supplement can be used to improve gastrointestinal health and psycho-physical conditions in dogs.

## 1. Introduction

Oxidative stress has been linked to the pathogenesis of many diseases and inflammatory conditions in both humans and animals. It results from a change in the cell’s redox balance brought on by an excess of reactive oxygen species (ROS) or a deficiency in the antioxidant system [1]. ROS are toxic by-products of oxygen metabolism well known for their high reactivity. The gastrointestinal (GI) tract is a critical site for the generation of pro-oxidants because it contains several bacteria and nutrients that interact with the immune system [2], and ROS are mostly produced by the GI tract [3] and can damage cells through the oxidation of some important molecules such as proteins, lipids, or DNA [4].

According to reports, oxidative stress contributed to the spread of various canine infections, including babesiosis, leishmaniasis, and ehrlichiosis [5]. Additionally, when compared to healthy subjects, the activities of antioxidant enzymes (catalase and superoxide dismutase) were different in dogs that had gastroenteritis brought on by parvovirus infection [5]. A redox imbalance was also observed in dogs with acute diarrhea [6]. Furthermore, oxidative stress leads to an increase in cytokines in the canine GI tract, which, in turn, increases the production of ROS [3]. Meanwhile, excess ROS are linked to gut dysbiosis as well as lipid peroxidation and cellular damage [7]. In the canine GI tract, infections and inflammations act like stressors, which results in an increase in ROS production. Studies have shown that elevated levels of ROS may play an important role in the development of acute enteropathies in dogs, causing mucosal injury and/or delaying recovery [6].

In the case of GI disorders in humans, oxidative stress plays a part in the emergence of GI [8] and stomach malignancies [9], as well as inflammatory bowel disease (IBD) [10]. In tissues from esophageal squamous cell carcinoma (ESCC), for example, oxidative stress markers are enhanced [11], while mineral and antioxidant vitamins are decreased, all of which contribute to ESCC [12]. In addition, factors that contribute to gastroesophageal reflux disease (GERD), such as acid, bile salts, and the consequent esophagitis, increase the production of ROS, leading to a general reduction in antioxidant levels, increased expression of ROS-inducible genes, and decreased levels of glutathione (GSH) and vitamin C, which are two important antioxidants. GERD is a known cause of esophageal adenocarcinoma.

The canine GI tract is inhabited by a complex microbiota that relates symbiotically with the host. The microbiota consists of fungi, bacteria, protozoa, and viruses, which correspond to microbial cells [13]. The gut microbiome does not remain constant. Instead, it varies due to changes in extrinsic or intrinsic factors such as age, diet, and reproductive conditions. Any alteration of the intestinal microbiota in dogs may lead to pathological events such as diarrhea, malabsorption, allergies, obesity, and stress [14,15,16,17]. Therefore, due to the gut–brain axis, stress affects the composition of the intestinal microbiota, increases the vulnerability to inflammatory stimuli in the GI tract, and has immunosuppressive effects [18]. In particular, dogs housed in kennels have greater susceptibility than dogs living with their owners. In fact, the chronic stress experienced in dog breeding centers, kennels, or shelters is mostly linked to changes in hierarchical relationships and social interactions between subjects, confined environments with spatial restrictions, repeated pregnancy, and changes in animal behavior. This situation predisposes dogs to intestinal disorders and inflammation [19,20,21,22].

In recent years, many researchers in both human and animal sciences have focused on the role played by oxidative stress in the etiology of acute and chronic intestinal disorders and inflammation and how to manage it. Consequently, research has been conducted on a variety of dietary antioxidant compounds in view of their use in the treatment and management of these conditions [23].

Specifically, antioxidant ingredients help to prevent oxidative damage, which, in turn, strengthens the immune system and gut microbiome [24]. Research suggests a strong mutualistic association between gut health and immune response in dogs [25], so alteration of the gut due to the use of antibiotics, an unbalanced diet, and other variables can influence the manifestation of various diseases in dogs [26,27]. Evidence of altered redox homeostasis has been reported in both acute and chronic enteropathies, and in individuals with simple enteropathy, the use of antioxidants has been proposed as a potential medication/drug substitute [28].

For example, the usefulness of antioxidant substances was found in *Lentinula edodes*, a mushroom that improves the intestinal barrier function, with the production of antimicrobial peptides characterized by high antioxidant properties, immunomodulation, and intestinal trophic effects in humans [29,30,31]. Bromelain extracts, which derive from pineapple and contain proteolytic enzymes with anti-inflammatory and immunomodulatory effects, are proven to provide digestive assistance, enhance wound healing, improve the cardiovascular and circulatory systems in humans, and help with gastric ulcers in animals [32]. Extracts of quercetin, a polyphenolic antioxidant substance, can stimulate the immune response in dogs [33]. In addition, quercetin, for example, has been proven to be effective in treating IBD [34], as well as colitis induced by trinitro-benzene sulfonic acid (TNBS), acetic acid, and dextran sodium sulfate (DSS) [35].

The aim of this study was to analyze the antioxidant capacity of three natural products (bromelain, quercetin, and *Lentinula edodes*) and develop a formulation for a feed supplement using a combination of the three substances. In addition, the evaluation of the effects of the feed supplement on dogs in kennel conditions was performed by measuring biomarkers of stress, inflammation, and intestinal metabolism.

## 2. Materials and Methods

### 2.1. In Vitro Study

#### 2.1.1. Materials

In this study, powdered samples of three natural products (bromelain (B), quercetin (Q), and *Lentinula edodes* (LE) mushroom) were used. They were obtained from a certified pharmaceutical company (Candioli srl, Beinasco, Italy). Bromelain was obtained from stems and fruits of pineapple (*Ananas comosus*) powder; quercetin was obtained from grape skins (*Vitis vinifera* ssp. *sativa*) powder; and mushroom was derived from *Lentinula edodes* mushroom powder. All these products belong to the category of feed materials [36,37]. The mixture was obtained using the same extract concentration for each individual substance when tested. The proximate chemical composition of the feed materials containing bromelain, quercetin, and *Lentinula edodes* has been added to the Supplementary Materials (Table S2).

#### 2.1.2. Chemicals and Reagents

Crude oil (ether extract), crude protein, and crude fiber were all analyzed using analytical-grade solvents that were acquired from Honeywell Fluka (Fisher Scientific Italia, Milan, Italy). Chemical standards (analytical grade) were provided by Sigma-Aldrich (Merck KgaA, Darmstadt, Germany). Sodium carbonate, potassium persulfate, 2,2′-Azino-bis (3-ethylbenzothiazoline-6-sulfonic acid) diammonium salt (ABTS), 6-hydroxy-2,5,7,8-tetramethy-chromam-2-carboxylic acid (Trolox), gallic acid, and 2,2-diphenyl-1-picrylhydrazyl (DPPH) were also purchased from Sigma-Aldrich (Merck KgaA, Darmstadt, Germany).

#### 2.1.3. Antioxidant Assays

*Sample Preparation and Extraction.* Organic solvent extractions were performed using a modified version of Fu et al.’s [35] method: 30 mL of 80% ethanol was used to treat 5 g of samples for 1 h in triplicate in a silicon bath at 60 °C. Following each extraction, the sample was filtered three times with 80% ethanol, washed, and diluted to 200 mL with this solvent. The three tested products were evaluated for their individual antioxidant activity and total phenolic content (TPC), as well as their combined antioxidant activity, using the extract that was thusly prepared. A total of 10 mL of each extract was combined to create the mixture. The final volume of 30 mL was reduced to a total of 10 mL by evaporation. The final concentrations of quercetin, bromelain, and *Lentinula edodes* were therefore the same in the single extracts and the mixture.

*Total Phenolic Content (TPC).* Following the procedure described by Karamać et al. [38], the TPC was assessed spectrophotometrically following the reaction with the Folin–Ciocalteu reagent (FCR) as follows: 250 μL of an extract dissolved in 80% ethanol (diluted 1:150 for quercetin), 250 μL of FCR, and 500 μL of sodium carbonate (10% solution), all combined with 4 mL of water. Then, incubation of the mixture was conducted in a dark place for 25 min at ambient temperature before being centrifuged for 5 min at 5000 rpm. A Cary 60 UV-Vis spectrophotometer was then used to measure the absorbance at a wavelength of 725 nm (Agilent Technologies, Santa Clara, CA, USA). For each gram of powdered sample, the results were represented as mg of gallic acid equivalents (GAE, mg/g). Each determination was made in triplicate.

*DPPH Free Radical Scavenging Assay.* DPPH assays were used to evaluate the antioxidant capacity of 80% ethanol extracts. According to the method outlined by Brand-Williams et al. [39], the DPPH scavenging activity was assessed. The DPPH radical is stable in ethanol solution; the DPPH radical was scavenged by antioxidant extracts, and the decrease in absorbance at a wavelength of 517 nm served as a measure of the level of DPPH radical reduction. To a final amount of 2.35 mL, various quantities of ethanolic extracts (from 10 µL to 200 µL) were added, along with 1 mM DPPH (0.25 mL) and 80% ethanol. The mixture was then mixed, and the absorbance was determined after 20 min using a Cary 60 UV-Vis spectrophotometer. The quantity (μg) of dried extract required to scavenge half of the DPPH was calculated using these data. The results were then represented as EC_50_ values, defined as the powdered extract concentration (in mg/mL of test product solution) required to scavenge 50% of the baseline DPPH.

*ABTS Radical Assay.* By using the ABTS technique, extracts of 80% ethanol were evaluated for their antioxidant potential. The technique described by Karamać et al. [38] was used to assess the Trolox equivalent antioxidant capacity (TEAC). A total of 2 mL of the ABTS diammonium salt radical cation was added to 20 μL of the 80% ethanolic solution of the extracts or the ethanolic Trolox standard solution. The mixture was vortexed and heated to 30 °C. Within 6 min, a Cary 60 UV-Vis spectrophotometer was used to read the absorbance at λ = 734 nm. The results were calculated and represented as µmol TE (Trolox equivalents)/g of the test product.

#### 2.1.4. Statistical Analysis—In Vitro Study

Data were subjected to a one-way ANOVA (*p* < 0.05). To ensure normality of data, percentage values were previously arcsine square root transformed. After ANOVA, Tukey’s test was applied to compare means using XLSTAT (v 2021.2.2, Microsoft Excel, Paris, France).

### 2.2. Feed Supplement Formulation

The new feed supplement formulation was based on extrapolations from the preliminary study and the literature data [33,40,41,42]. For safety reasons, the dosage in mg/Kg BW of each of the three natural products included in the new supplement was lower than the maximum dosage reported in these trials. The ingredients used in the new supplement are listed in Table 1.

### 2.3. In Vivo Study

#### 2.3.1. Animals and Study Design

American Staffordshire Terrier adult female dogs (5 ± 1 years old, weighing 17 ± 1.5 kg) were chosen from an ENCI (Ente Nazionale Cinofilia Italiana)-registered breeder in Italy for this double-blinded randomized control study. A written informed consent form was signed by the dog breeder after being apprised of this study’s procedures. Regulations for the care and use of animals set forth by the Italian Ministry of Health were followed during this study’s execution (D.L. n. 26, 2014), and Regulation (EC) N. 767/2009 was used to regulate the use of supplements. This study was approved by the Ethical Animal Care and Use Committee of the University of Turin (19 July 2022; protocol n. 2741).

A total of 30 healthy dogs were randomly assigned into two groups: control (CTR, *n* = 15) and treated (TRT, *n* = 15). Dogs were kept in groups of three dogs per box. Each box measured 6 (±2) meters squared to ensure animal welfare principles and to exclude social stress resulting from collective manipulation. Boxes were selected with an open space. A commercial diet (Royal Canin Medium Adult) was supplied to both groups before the start of the experiment by at least 7 days. The following equation was used to calculate the daily calorie intake:

ME (kcal/day) = 110 kg BW ^0.75^

The CTR and TRT groups were supplemented with the placebo (Table 1) and the newly formulated feed supplement (Table 1), respectively, as powder in their food once daily for a total of 28 days (T0 to T28). The dosage used was set as 1 mg every 10 Kg of BW.

The veterinarian at the beginning of the investigation (T0) checked the health status of the animals via a general physical examination and a fecal analysis to ensure that no underlying conditions were present.

#### 2.3.2. Nutritional Parameters

The same veterinarian recorded the animals’ body weight (BW) at time zero (T0) and time 28 (T28) of the experiment. Each animal was examined visually and palpated by the same knowledgeable veterinarian at T0 and T28 to obtain a Body Condition Score (BCS), which ranges from 1 to 9 (a score of 4 or 5 is the desired outcome (WSAVA, 2013a)) [43].

#### 2.3.3. Fecal Parameters

Calprotectin, cortisol, N-methylhistamine (NMH), putrefactive fecal compound (indole/skatole), and short-chain fatty acid (SCFA) concentrations were measured using fresh feces in laboratory analysis. Additionally, the pH was determined.

The breeder collected fresh excrement with a spatula every week in the morning and placed it in a sterile plastic bag, then in a recording box with an assigned dog code. The samples were maintained at 4 °C and taken to the laboratory. Fecal analyses were performed at the start of the trial (T0), then 7, 14, 21, and 28 days later (T7, T14, T21, and T28, respectively). Following a blinded sample-identification protocol, the analysis was performed by the same laboratory technician.

An enzyme-linked immunosorbent assay (ELISA) manufactured and analytically validated at Texas A&M University’s gastrointestinal laboratory specifically for measuring fecal calprotectin concentration was used. Spot fecal samples (1.0 ± 0.3 g) were collected and stored frozen (−20 °C) for 2–20 months until analysis. Fecal samples were thawed and extracted, and calprotectin biomarker concentration was measured in 2 batches of all specimens using an ELISA kit (Bühlmann Laboratories AG, Schönenbuch, Switzerland). To prepare the fecal samples, approximately 100 mg of each sample was homogenized in 5 mL of extraction buffer as per the manufacturer’s instructions. Next, 2 mL of the homogenate was subjected to centrifugation in a micro-centrifuge at 250 rpm for 5 min. The resulting supernatant was diluted (1:50) with incubation buffer, and 3000 g plus 100 μL of the diluted supernatant was then incubated at room temperature (25 °C) and loaded onto a microtiter plate that was coated with a highly specific monoclonal capture antibody for calprotectin heterodimeric and polymeric complexes. Following an incubation period of three days, the samples were washed with a phosphate saline solution (pH 8) and then subjected to further incubation. A further washing step with a phosphate saline solution (pH 8) was performed, and an aliquot of tetramethyl benzidine (5 μL) was added to stop the reaction. The optical density was immediately measured at 450 nm using a microplate spectrophotometer reader.

A cortisol Enzyme Immunoassay (EIA) was used to analyze fecal extracts. Cortisol standards (hydrocortisone; Sigma-Aldrich, St. Louis, MO, USA) and a cortisol-horseradish peroxidase (Cortisol—HRP) ligand and antiserum (No. R4866; CJ Munro, University of California, Davis, CA, USA) were used in the experiment. The polyclonal antibody was developed in rabbits against the cortisol-3-carboxymethyl oxime linked to bovine serum albumin and exhibits cross-reactivity with cortisol at 100%, prednisolone at 9.9%, cortisone at 5%, and androstenedione, androsterone, and 11-desoxycortisol at 1%. A 96-well microtiter plate (Nunc-Immuno, Maxisorp Surface; Fisher Scientific, Pittsburgh, PA, USA) was used to perform the EIA. The plate had previously been coated with cortisol antiserum (14–18 h):(50 μL/well; diluted 1:20,000 in coating buffer; 0.05 M NaHCO_3_, pH 9.6). Steroid buffer (0.1 M NaPO_4_, 0.149 M NaCl, pH 7.0) was used for dilution of fecal extracts after they were evaporated to dryness, and then they were assayed in duplicate. Cortisol standards (50 μL, range 3.9–1000 pg/well, diluted in assay buffer, 0.1 M NaPO_4_, 0.149 M NaCl, 0.1% bovine serum albumin, pH 7.0) and samples (50 μL) were combined with cortisol-horseradish peroxidase (50 μL, 1:8500 dilutions in assay buffer). Incubation was performed at room temperature for 1 h, and then to each well, 100 μL substrate buffer (0.4 mM 2,2′-azino-di- (3-ethylbenzthiazoline sulfonic acid) diammonium salt, 1.6 mM H_2_O_2_, 0.05 M citrate, pH 4.0) was added after washing the plates 5 times. The absorbance was measured at 405 nm after incubation on a shaker for 10–15 min. Parallel displacement curves for each species were discovered by contrasting the serial dilutions of pooled fecal extracts with the cortisol standard preparation. The coefficients of variation within an assay (*n* = 26 duplicates of a single sample) and between assays (*n* = 57 assays) were 6.4% and 11.0%, respectively. The assay’s sensitivity was 3.9 pg/well at maximal binding.

Stable isotope dilution gas chromatography (GC-MS) was used for measuring N-methylhistamine (NMH). Briefly, 50 pg of tri-deuterated NMH was added as an internal standard to 200 µL of each fecal extract (dilution 1:5). Then sodium borate buffer (pH 9, 10 mM, 200 µL) was added. The sample was applied to a solid-phase silica extraction column after it was subjected to vortexing. Changes in chromatography-grade water were used for washing the columns, and then 0.1 N HCl-acidified methanol was used for eluting the sample. A heating block was used for the evaporation of the eluted samples to dryness using nitrogen. Methanol in chloroform (300 μL of a 20% solution) was used for reconstitution of the dried sample before application to the second solid-phase silica extraction column. Methanol in chloroform (150 μL of a 20% solution) was used for washing the column. Four 1 mL volumes of methanol: chloroform: ammonium hydroxide (25:25:1, *v*/*v*) were used for eluting the sample and dried as described earlier. To make derivatization, 200 µL of ethyl acetate, 40 µL of pyridine, and 100 µL of pentafluoro propionic anhydride were added to the sample and incubated for 40 min at 64 °C. In the next partitioning step, 500 µL of 0.5 M Tris buffer was added to each sample, followed by 1.5 mL of hexane. Vortex was applied to the samples for 1 min, and then they were centrifuged for 1 min at 574× *g*. The upper layer (hexane layer) was collected, another 1.5 mL of hexane was added to each sample, and the process was repeated. The 2 hexane fractions were combined and evaporated to dryness. The residue was reconstituted with 30 µL of ethyl acetate and vortexed before transfer to a gas chromatography mass spectrometry autosampler vial. A gas chromatograph and mass selective detector with a dimethylpolysiloxane capillary column were used to perform the gas chromatography—MS analysis; all other conditions (carrier gas, temperature, pressure, and gradient) used were similar to what has been described in the earlier fecal NMH assay in dogs. To evaluate the assay performance, a standard curve from 0 to 5000 pg/μL was processed prior to each run. NMH and deuterated isotopes were quantified by using the ions at an *m*/*z* of 417 and 420, respectively. Fecal concentrations of NMH were back-calculated for the wet weight of the sample and reported as nanograms per gram of faces.

Indole and skatole were extracted in accordance with Flickinger et al. (2003) [44]. Briefly, 2 g of faces was combined with 5 mL of methanol, parafilm was used to cover the mixture, and the mixture was incubated for 1 h at 4 °C while being constantly agitated. As previously described by Flickinger et al. (2003) [44], the supernatant was removed after centrifuging at 2124× *g* for 10 min at 4 °C. Then, 5 mL of methanol was added to the pellet, mixed completely, and stored for 1 h. A combination of both supernatants was then analyzed for indole and skatole using a gas chromatograph. The internal standard was 5-chloro indole. The initial temperature of the inlet was 200 °C, and injection was in splitless mode. The initial temperature of the oven was 85 °C, maintained for 2.0 min, and the temperature program included an increase of 10 °C per min until 250 °C, maintained for 4.0 min. The carrier gas was helium, and the FID (Flame-Ionization Detector) temperature was 220 °C.

The concentrations of SCFAs were measured in the fecal samples using a GC-MS assay. The fecal samples were initially weighed and diluted in a 2N hydrochloric acid extraction solution and then frozen at −80 °C until analysis for up to 3 months. After thawing, the fecal suspensions were homogenized by a multitube vortexer at room temperature for 30 min and then centrifuged for 20 min at 2100× *g* and 4 °C. Supernatants were collected using serum filters, and 500 μL of each sample was mixed with 10 μL of an internal standard (200 mM heptadeuterated butyric acid) and extracted using a C18 solid-phase extraction column. The samples were then derivatized using N-test-butyldimethylsilyl-N-methyltrifluoroacetamide (MTBSTFA) at room temperature for 60 min. Chromatographic separation and quantification of the derivatized samples were performed using a GC coupled with an electron ionization MS, with separation achieved using a DB1 ms capillary column. The MS was operated in electron impact positive ion mode with selective ion monitoring at mass-to-charge ratios (*m*/*z*) of 117 (acetate), 131 (propionate), 145 (butyrate), and 152 (heptadeuterated butyrate; internal standard). Quantification was based on the ratio of the area under the curve of the internal standard to each of the fatty acids. Final concentrations of fecal SCFAs were adjusted by fecal dry matter (DM) and expressed as μmol/g of fecal DM to account for differences in water content between fecal samples.

Finally, before measuring the pH, the samples were kept at room temperature (20–23 °C) and stirred for one minute. Then, the pH levels were recorded using a calibrated pH instrument (HI 9125 pH/ORP meter; Hanna Instruments, Bedfordshire, UK).

#### 2.3.4. Statistical Analysis—In Vivo Study

The statistical analysis of the fecal sample data was conducted using RStudio integrated development environment (Version 1.4.1717, R Foundation for Statistical Computing, Boston, MA, USA) [45]. To confirm the normality of the data distribution, the Shapiro–Wilk test was performed. The laboratory data, including calprotectin, cortisol, SCFA, indole/scatole, N-methylhistamine, and pH, were used as outcomes in linear mixed models where treatment, time, and treatment-by-time interaction were included as fixed-effect predictors. A random intercept term was included to consider repeated measurements on the same dogs. The lmer function in the lme4 package was used for this analysis (Model Summary Table available in the Supplementary Material: Table S1). Bar charts and box plots were created using the ggplot2 and ggpubr packages to visualize the means, standard error means, and pairwise t-tests between treatment groups at each time point. The threshold for statistical significance was set at *p* < 0.05.

## 3. Results

### 3.1. In Vitro Study

The TPC, DPPH, and ABTS are common methods to determine the antioxidant activities of substances. These methods work based on the metal-chelating properties and scavenging abilities of radicals [46].

The three powdered extracts containing bromelain, quercetin, and *Lentinula edodes* had TPC mean values of 4.42, 1.68, and 2.99 (mg GAE/g DM), respectively. Then, the mixture of all three substances had a TPC of 4.34 (mg GAE/g DM).

The results of the DPPH analysis indicated that, on average, EC_50_ values of 433.61, 0.82, and 230.0 µg/mL were exhibited by the individual substances of bromelain, quercetin, and the *Lentinula edodes* mushroom, respectively. A value of 137.57 µg/mL was registered for the mixture. The ABTS values were 17.55 for bromelain, 10.73 for quercetin, and 21.71 (µmol TE/g DM) for the mushroom. The mixture displayed a value of 124.91 (µmol TE/g DM). Table 2 shows the total phenolic content and the antioxidant capacity of the single substances and the mixture. The mixture and bromelain showed significantly higher TPC mean values than *Lentinula edodes* and quercetin; the latter showed the significantly lowest TPC content. Nevertheless, quercetin presented the significantly lowest mean value for DPPH, followed by the mixture, *Lentinula edodes*, and bromelain; all the substances showed significant differences between each other. The mean ABTS value of the mixture was significantly higher than that of the three individual substances, which did not differ significantly among each other (*p* > 0.05).

### 3.2. In Vivo Study

All dogs in the experiment retained their normal health throughout the whole experiment without any undesirable or side effects. No food waste was found in any of the stalls throughout the period. There was no change in food consumption. Of interest, there was no significant difference (*p* > 0.05) in BW and BCS between the initial phase (T0) and the final phase of the experiment (T28). The animals did not show any significant differences (*p* > 0.05) at the start of the experiment for any of the fecal parameters analyzed.

The linear mixed models resulted in significant treatment*time interactions for all the response variables (fecal calprotectin, cortisol, indole/skatole, SCFA, and N-methylhistamine) except pH, as shown in Table 3. Such interactions indicated that the changes in time in these variables were different in the TRT group as compared to the CTR group, demonstrating a significant effect of the treatment on the dogs. Specifically, in the treated subjects, fecal calprotectin, cortisol, indole/skatole, and N-methylhistamine decreased over time, whereas there was an increase in SCFA levels. Conversely, in the control subjects, there was no change in these variables over time.

The treatment resulted in a significant gradual decrease in calprotectin levels to 75%, 68%, and 55% at T14, T21, and T28, respectively, as compared to the control groups at each time point (*p* < 0.001). Similarly, the treatment induced a significant reduction in cortisol levels to 91% at T7 (*p* < 0.01), 81%, 68%, and 64% at T14, T21, and T28 (*p* < 0.001), respectively, as compared to the control groups at each time point. In a similar way, the treated groups exhibited significant depressed indole/skatole levels of 80%, 69%, 63%, and 51% at T7, T14, T21, and T28, respectively (*p* < 0.001), as compared to the control groups at each time point. The N-methylhistamine level was decreased significantly by the treatment to 94% at T7 (*p* < 0.01), 79%, 72%, and 60% at T14, T21, and T28, respectively (*p* < 0.001), as compared to the control groups at each time point. In contrast, the treatment induced a significant gradual elevation in SCFA levels of 1.10, 1.25, 1.32, and 1.40 times at T7, T14, T21, and T28, respectively (*p* < 0.001), as compared to the control groups at each time point (Table 3). There was not any significant change in pH at any time point in the CTR and TRT groups (Table 3). A graphical representation of the effects of the supplement can be found in the Supplementary Materials (Figure S1).

## 4. Discussion

Free radicals and oxidative stress are factors associated with the onset and progression of a number of conditions in both humans and animals, including GI, respiratory, and neurological disorders [2,47]. In this study, the antioxidant activities of three natural products (bromelain, quercetin, and *Lentinula edodes*) and their combination have been evaluated in the laboratory and in dogs in kennel conditions. Beside the fact that the three extracts (bromelain, quercetin, and *Lentinula edodes*) used individually have already been tested in vivo for their effects on GI disorders [31,48,49], no study has reported the use of these three combined.

TPC and antioxidant activity are directly correlated, with increased TPC corresponding to higher antioxidant activity [49]. Several factors are responsible for the variable amount of TPC in a sample. The major contributors to antioxidant activity are flavonoids, which are phenolic compounds. In the literature, *Lentula edodes* was reported to contain a variable amount of total phenolics (0.4 mg/g to 3.4 mg/g), with slight differences in values depending on various conditions such as medium of growth and mode of testing [50]. According to Arrabi et al. [51], quercetin is mainly composed of flavonoids, which are also phenolics. The phenolic compounds are also reported to be the major source of TPC in phenols [52]. Considering our TPC analysis, the mixture had the highest value but was not significantly different from the bromelain one. This could reflect the antioxidant effect of bromelain in the mixture, with no negative impact of the other substances on the mixture’s properties.

For the DPPH assay, the lower the value, the greater the antioxidant capacity. The DPPH analysis results showed that quercetin has the highest antioxidant potential among the individually tested samples. Similar results were reported by Hirano et al. in [53], where quercetin was found to be the best metabolite compared to other metabolites such as myricetin, luteolin, and apigenin, and only 3 μM of an ethanol extract of quercetin was used to scavenge 50% of the DPPH radicals. This indicates relatively good antioxidant properties for this ingredient. Quercetin is a dietary flavonoid demonstrated to have good antioxidant properties that has been commonly used in studies on flavonoids and their antioxidant activity [46].

The ABTS analysis of the three substances revealed consistent results in the range of 10 to 21 µmol TE/g DM. The possible cause of these little variations could be the mechanism of antioxidant activity and electron transfer from phenolate ions, as already stated in the literature [54]. For example, Montserrat and colleagues (2011) reported the antioxidant activity of quercetin to be 7.52 µmol TE/g DM by ABTS assay [46], and this result is very similar to ours. Interestingly, our mixture displayed a significantly higher value compared to the individual substances. The mixture showed a significantly higher mean ABTS value compared to all three individual substances, the highest TPC mean values together with bromelain, and a good value for DPPH.

Given the promising results obtained from the laboratory analysis and the good antioxidant potential of the mixture under test, the use of this supplement in dogs in kennel conditions would be beneficial for their well-being, especially for their gut health. In fact, it has been reported in the literature that dog-kennel conditions may result in a higher susceptibility to gastrointestinal disorders compared to dogs that live with their owners. This is likely due to the chronic stress to which they are subjected, which is linked to changes in hierarchical relationships and social interactions between subjects, as well as confinement to restricted environments with repeated pregnancies and subsequent changes in animal behavior [19,20,21,22]. Because of the gut–brain axis, stress can alter the composition of the intestinal microbiota, thereby enhancing the susceptibility of the gastrointestinal tract to inflammatory stimuli, causing dysbiosis, and eliciting immunosuppressive effects [18].

In this study, the use of fecal parameters was very helpful to show changes in the animals’ stress status and GI health. In addition, collecting fecal samples has the advantage of creating less stress compared to proceeding with a blood sample through venipuncture.

In particular, fecal calprotectin, which is a calcium and zinc-binding heterodimeric complex consisting of S100A8 and S100A9 proteins [55,56], was used as a stool non-invasive biomarker. Calprotectin exists in neutrophils, monocytes, and reactive macrophages in stool. Fecal calprotectin concentrations are good indicators for clinical disease activity in a direct correlation, which is confirmed by the endoscopic and histologic scoring of biopsy specimens [57,58,59]. At the beginning of the current study, the level of calprotectin for both groups was within normally accepted limits as described in the literature [60]. The administration of the feed supplement resulted in a significant gradual decrease in calprotectin levels starting from day 14 up until the end of the present study (day 28) as compared to the control group. The current results are in line with a study carried out on a colitis-induced model in Wistar rats, which demonstrated the significant effect of quercetin nanoparticles in decreasing fecal calprotectin levels [61]. This is also in line with a human clinical study where the use of a mushroom (*Agaricus blazei* Murill) resulted in decreased fecal calprotectin levels in patients with IBD (ulcerative colitis in particular) [62]. Elevated levels of fecal calprotectin indicate neutrophil migration into the intestinal mucosa following intestinal injury, and the effect of quercetin on decreasing calprotectin levels may be attributed to its antioxidant effect [61].

Regarding cortisol, this is an indicator of stress in dogs, and its levels are increased after exercise and training in outdoor conditions [63]. It was documented that sheltered free-roaming dogs express higher plasma cortisol and fecal cortisol metabolites than domestic pets, also suggesting a direct correlation between plasma cortisol and fecal cortisol [64]. When pets are exposed to restraint conditions, they experience psychological stress with higher levels of cortisol due to activation of the hypothalamus–pituitary–adrenal and sympathetic–adrenal medullas with prolonged sustained oxidative damage [65]. In our study, the fecal cortisol levels recorded gradually decreased from day 7 to the end (day 28) as compared to the control group. This favorable decreasing effect of feed on cortisol levels may be due to the antioxidant effect elicited, which is also in line with a study that used a mixture of antioxidants (quercetin, resveratrol, curcumin, and vitamin E) in hyperthyroid cats [65].

SCFAs are the primary end products of the bacterial fermentation of non-digestible dietary fibers. SCFAs exert an anti-diarrheic, immunomodulatory, and regulatory effect on the motility of the gastrointestinal tract [43]. In clinical studies, patients with IBD presented with lower concentrations of SCFAs [66]. In our study, dogs in the TRT group exhibited significantly higher levels of SCFAs as compared to the ones in the CTR group. The current results are in line with a study performed in an osteoarthritis rat model in which quercetin succeeded in increasing the fecal level of SCFAs, which may be related to the antioxidant and anti-inflammatory effects of quercetin [67]. The results of our study suggest that the administration of a natural feed supplement may promote a balanced gut microbiota and enhance the gastrointestinal health of dogs. The significant increase in fecal SCFA levels in dogs that received the supplement may be attributed to the fermentation of non-digestible dietary fibers by the gut microbiota [68], leading to the production of SCFAs.

Histamine is an important mediator of many physiological and pathological functions [69]. An increased number of mast cells in the gastrointestinal tract of dogs with chronic enteropathies suggests a possible contribution of histamine release to the pathogenesis of canine inflammatory bowel disease [70]. In the present study, the administration of the natural feed supplements resulted in a significant decrease in the fecal N-methylhistamine level in comparison to the untreated dogs. This is in agreement with an old study, which reported that quercetin decreased the release of histamine from isolated dog immune cells [71]. Some canine supplements use quercetin to relieve allergies and use it as a natural antihistaminic agent that can heal itching [72].

Indole/skatole were reported to exert toxic effects on the intestinal mucosa [43], which were generated by the bacterial degradation of endogenous and undigested protein [41]. In the present study, the administration of the new feed supplement resulted in a significant decline in the fecal indole/skatole levels in comparison to the untreated dogs. One possible hypothesis for this observed effect is that the bromelain component in the supplement may have contributed to this reduction. Previous studies have reported that bromelain has anti-inflammatory and antimicrobial properties, and it was also reported to elicit an anti-inflammatory response by reducing the synthesis of prostaglandin E2 and cyclooxygenase-2 [42,73]. This can help regulate the gut microbiota and reduce the production of harmful bacterial metabolites, including indole/skatole. Therefore, it is possible that the bromelain component in the tested feed supplement may have played a key role in reducing the fecal indole/skatole levels. However, further research is needed to confirm this hypothesis and investigate the underlying mechanisms of action.

In the present study, no significant change in stool pH was recorded throughout the whole study, suggesting that the natural feed supplement did not exert any adverse impact on the gut environment of dogs. Maintaining a stable pH in the gut is critical for the health and well-being of dogs. A stable pH in the gut creates an environment that supports the growth of beneficial bacteria while inhibiting the proliferation of harmful pathogens. This finding has significant implications for the potential use of the new feed supplement to promote gastrointestinal health in dogs.

Maintaining stable BW and BCS throughout the whole study reflects the potential impact of the used feed supplement on maintaining a good health status. The BCS is considered a very valid and effective indication for body fat; a higher BCS may promote opportunistic pathogens and suppress some beneficial bacteria [74].

One potential limitation of this study is its exclusivity to female dogs. In future studies, it would be beneficial to include both male and female dogs to enhance the generalizability of the findings. Additionally, considering the symbiotic relationship between the gut microbiota and the host, it is important to acknowledge that the composition of the gut microbiota can be influenced by various external factors, such as changes in dietary patterns. Therefore, it would be valuable to explore the effects of the supplement on the composition of the gut microbiota as well. This would provide a more comprehensive understanding of how the supplement interacts with the gut microbiota and its potential implications.

## 5. Conclusions

In this study, three natural substances (quercetin, bromelain, and *Lentinula edodes*) tested individually showed variable antioxidant capacities, but their mixture showed promising strong effects as an antioxidant. A new formulation based on this mixture was used in dogs in kennel conditions, resulting in a significant improvement in gut health. The decrease in fecal calprotectin, cortisol, indole/skatole, and N-methylhistamine levels suggests a decrease in the inflammatory status and enhancement of gut microbiota composition in the treated dogs. Additionally, the increase in SCFA levels indicates enhanced fermentation of dietary fibers, which may exert anti-inflammatory and immunomodulatory effects on the gastrointestinal tract. These findings support the potential use of this new natural feed supplement as a dietary intervention to improve gastrointestinal health and psycho-physical conditions in dogs. Further studies are needed to understand the effects on animal health over a longer period of time, on different age groups and breeds, and in animals affected by GI disorders.

## Figures and Tables

**Table 1:** List of the ingredients for the new feed supplement and for the placebo.

New Formulation Ingredients	mg/g
*Lentinula edodes*	10.0
Quercetin	13.5
Bromelain	13.5
Maltodextrin	583.4
Appetite stimulants	379.6
Total	1000.0
**Placebo ingredients**	
Maltodextrin	1000.0

**Table 2:** Total phenolic content and antioxidant capacity in single substances and mixture.

Assays Performed	Bromelain (B)	Quercetin (Q)	*Lentinula edodes* (LE)	Mixture (B + Q + LE)
TPC (mg GAE/g DM)	4.42 a	1.68 c	2.99 b	4.34 a
DPPH (EC_50_ µg/mL)	433.61 a	0.82 d	230.90 b	137.57 c
ABTS (µmol TE/g DM)	17.55 b	10.73 b	21.71 b	124.91 a

TPC: Total phenolic content, expressed as gallic acid equivalents (GAE mg/g dry matter); DPPH: 2, 2-diphenyl-1-picrylhydrazyl scavenging activity, expressed as EC_50_ (concentration of dried extract mg/mL solution); ABTS: 2,2′-Azino-bis (3-ethylbenzothiazoline-6-sulfonic acid) diammonium salt scavenging activity, expressed as µmol TE (Trolox equivalents)/g dry matter). Mean values followed by different letters (a, b, c, d) within the same row differ significantly for *p* < 0.0001.

**Table 3:** Mean (± S.E.M) values of fecal parameters at each time point (T0-T28) in the treated (TRT) and control (CTR) groups. Effects of treatment and time interaction on the fecal parameters in the TRT group.

Time	Group	Fecal Parameter					
		Calprotectin (µg/g) Mean ± S.E.M	Cortisol (pg/mg) Mean ± S.E.M	Short-chain fatty acids (μmol/g) Mean ± S.E.M	Indole/scatole (μmol/g) Mean ± S.E.M	N-methylhistamine (ng/g) Mean ± S.E.M	pH Mean ± S.E.M
**T0**	CTR	5.75 ± 0.18	0.62 ± 0.01	199.9 ± 1.0	1.68 ± 0.06	108.5 ± 1.6	6.51 ± 0.06
	TRT	5.79 ± 0.26	0.63 ± 0.01	200.0 ± 0.7	1.69 ± 0.08	109.6 ± 1.4	6.52 ± 0.04
	*p*-value	0.9	0.7	>0.9	0.9	0.6	>0.9
**T7**	CTR	5.76 ± 0.18	0.65 ± 0.01	199 ± 1	1.86 ± 0.06	110 ± 2	6.47 ± 0.06
	TRT	5.22 ± 0.28	0.59 ± 0.02	218 ± 4	1.48 ± 0.05	104 ± 2	6.48 ± 0.06
	*p*-value	0.12	0.003	<0.001	<0.001	0.007	>0.9
**T14**	CTR	5.88 ± 0.19	0.63 ± 0.01	197 ± 3	1.78 ± 0.07	112 ± 1	6.48 ± 0.06
	TRT	4.43 ± 0.23	0.51 ± 0.01	247 ± 4	1.23 ± 0.05	89 ± 3	6.49 ± 0.05
	*p*-value	<0.001	<0.001	<0.001	<0.001	<0.001	0.9
**T21**	CTR	5.81 ± 0.20	0.65 ± 0.01	198 ± 3	1.75 ± 0.05	111 ± 2	6.51 ± 0.07
	TRT	3.92 ± 0.15	0.44 ± 0.01	259 ± 4	1.11 ± 0.08	80 ± 3	6.48 ± 0.04
	*p*-value	<0.001	<0.001	<0.001	<0.001	<0.001	0.7
**T28**	CTR	5.72 ± 0.18	0.63 ± 0.01	199 ± 1	1.80 ± 0.06	111 ± 2	6.52 ± 0.07
	TRT	3.14 ± 0.20	0.40 ± 0.01	270 ± 4	0.91 ± 0.03	67 ± 1	6.45 ± 0.06
	*p*-value	<0.001	<0.001	<0.001	<0.001	<0.001	0.5
Treatment * time interaction effect on each fecal parameter		−0.22 ***	−0.02 **	4.85 **	−0.06 **	−3.57 ***	−0.01

*** *p* < 0.001; ** *p* < 0.01; * *p* < 0.05.

## Data Availability

Data available upon request to the first author.

## Supplementary Materials

The following supporting information can be downloaded at: https://www.mdpi.com/2306-7381/10/8/486/s1, Figure S1: Bar charts comparative analysis (t-test) between treated (TRT) group and control (CTR) group at different time points; Table S1: Linear Mixed Models output summary: Effects of Treatment, Time and Treatment*Time interaction on fecal parameters; Table S2: Analytical chemical composition of feed products containing bromelain, Lentinula edodes and the mixture.
