# Correction: Atuahene et al. A Supplement with Bromelain, *Lentinula edodes*, and Quercetin: Antioxidant Capacity and Effects on Morphofunctional and Fecal Parameters (Calprotectin, Cortisol, and Intestinal Fermentation Products) in Kennel Dogs. *Vet. Sci.* 2023, *10*, 486

**DOI:** 10.3390/vetsci11040149

**Published:** 2024-03-27

**Authors:** David Atuahene, Annalisa Costale, Elisa Martello, Alessandro Mannelli, Elisabetta Radice, Davide Giuseppe Ribaldone, Biagina Chiofalo, Bruno Stefanon, Giorgia Meineri

**Affiliations:** 1 Department of Veterinary Sciences, School of Agriculture and Veterinary Medicine, University of Turin, 10124 Grugliasco, Italy; david.atuahene@unito.it (D.A.);; 2 Department of Science and Pharmaceutical Technology, University of Turin, 10100 Turin, Italy; 3 Division of Epidemiology and Public Health, School of Medicine, University of Nottingham, Nottingham NG5 1PB, UK; 4 Department of Surgical Sciences, Medical School, University of Turin, 10100 Turin, Italy; 5 Department of Medical Sciences, University of Turin, 10100 Turin, Italy; davidegiuseppe.ribaldone@unito.it; 6 Department of Veterinary Sciences, University of Messina, 98122 Messina, Italy; 7 Department of Agrifood, Environmental and Animal Science, University of Udine, 33100 Udine, Italy

## Text Correction

There was an error in the original publication [1]. In Section 2.3.1: Animals and Study Design, third paragraph, “The dosage used was set as 1 mg every 10 Kg of BW”, this should be corrected to “The dosage used was set as 1 g/10 kg of BW”.

A correction has been made to Section 2.3.1, page 5. The authors state that the scientific conclusions are unaffected. This correction was approved by the Academic Editor. The original publication has also been updated.
