# Supplementary material for: A Supplement with Bromelain, Lentinula edodes, and Quercetin: Antioxidant Capacity and Effects on Morphofunctional and Fecal Parameters (Calprotectin, Cortisol, and Intestinal Fermentation Products) in Kennel Dogs
Source: Vet Sci. 2023 Jul 27;10(8):486. doi: 10.3390/vetsci10080486 (PMC10459215; doi:10.3390/vetsci10080486)
Supplement: Supplementary file 1 [file vetsci-10-00486-s001.zip › vetsci-2443979-supplementary.pdf]

Supplementary Materials

Table S1. Linear Mixed Models output summary: Effects of Treatment, Time and Treatment*Time interaction on fecal parameters.

	**Calprotectin Model**	**Cortisol Model**	**Short Fatty Chain Acids Model**	**Indole/Scatole Model**	**N-methylhistamine Model**	**pH Model**
(Intercept)	5.78***	0.63***	199.28***	1.73***	109.07***	6.48***
	(0.28)	(0.02)	(5.14)	(0.08)	(3.25)	(0.06)
Treat	-0.46	-0.06*	22.13***	-0.27**	-7.65	0.02
	(0.38)	(0.02)	(6.31)	(0.09)	(4.10)	(0.08)
Time	-0.00	0.00	-0.22	0.01	0.47	0.01
	(0.05)	(0.00)	(1.41)	(0.02)	(0.82)	(0.01)
Treat:Time	-0.22***	-0.02**	4.85**	-0.06**	-3.57***	-0.01
	(0.06)	(0.01)	(1.52)	(0.02)	(0.90)	(0.01)
AIC	375.28	-341.30	1299.76	71.37	1155.15	-114.45
BIC	393.34	-323.23	1317.83	89.44	1173.22	-96.38
Log Likelihood	-181.64	176.65	-643.88	-29.69	-571.58	63.22
Num. obs.	150	150	150	150	150	150
Num. groups: Dog	30	30	30	30	30	30
Var: Dog (Intercept)	0.74	0.00	70.64	0.01	46.71	0.04
Var: Residual	0.39	0.00	296.49	0.07	101.92	0.01

$^{***}p < 0.001$; $^{**}p < 0.01$; $^{*}p < 0.05$. Model Formula: Treatment + Time + Treatment * Time + (1|Dog).

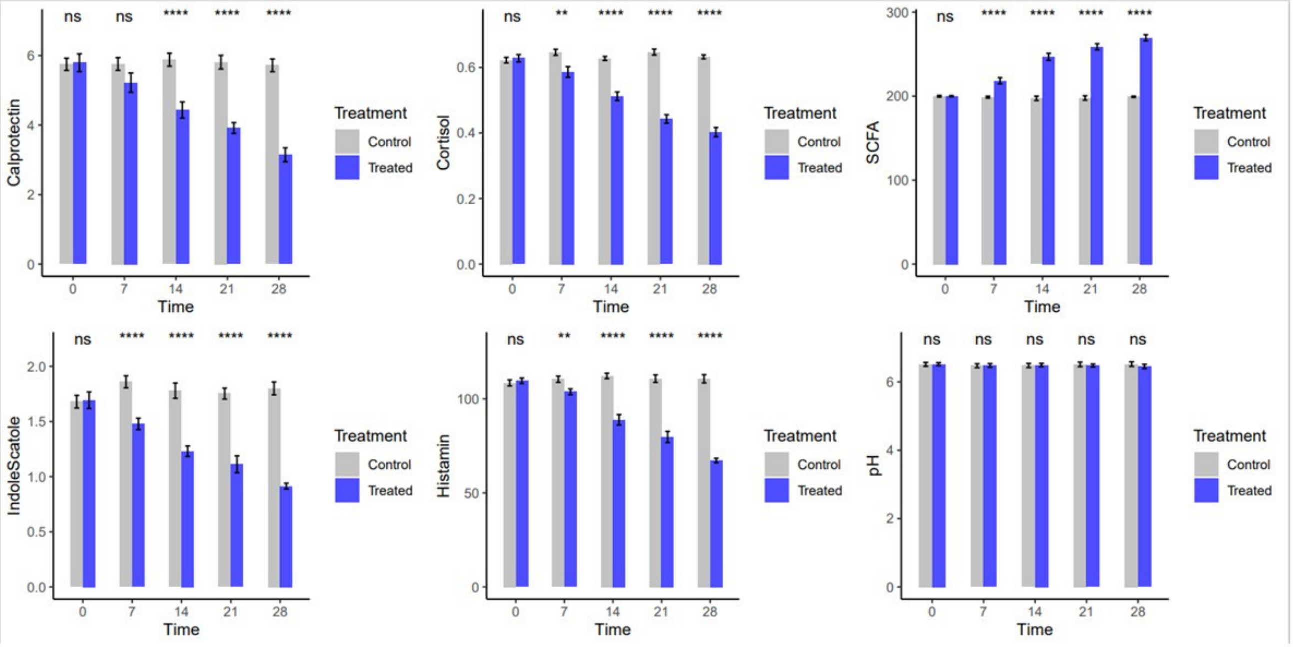

Figure S1. Bar charts comparative analysis (t-test) between treated (TRT) group and control (CTR) group at different time points. $^{***}p < 0.001$; $^{**}p < 0.01$; $^{*}p < 0.05$.

Table S2. Analytical chemical composition of feed products containing bromelain, Lentinula edodes and the mixture.

**Substances**	**Proteins (%)**	**Fats (%)**	**Ash (%)**	**Fiber (%)**
Bromelain (B)	2.13	0.10	8.42	3.42
Quercetin (Q)	0.3	45.86	5.89	0.5
Lentinula edodes (LE)	18.0	0.5	1.3	5.2
Mix (B+Q+LE)	9	4.6	5.4	3.5
